# Potential mode of action of multispecies inoculums on wheat growth under water stress

**DOI:** 10.1093/ismeco/ycaf095

**Published:** 2025-06-09

**Authors:** Asmaâ Agoussar, Julien Tremblay, Étienne Yergeau

**Affiliations:** Centre Armand-Frappier Santé Biotechnologie, Institut National de la Recherche Scientifique, 531 Boulevard des Prairies, Laval, QC H7V 1B7, Canada; Centre Armand-Frappier Santé Biotechnologie, Institut National de la Recherche Scientifique, 531 Boulevard des Prairies, Laval, QC H7V 1B7, Canada; Centre Armand-Frappier Santé Biotechnologie, Institut National de la Recherche Scientifique, 531 Boulevard des Prairies, Laval, QC H7V 1B7, Canada

**Keywords:** drought, inoculum, wheat, microbial communities, soil, rhizosphere

## Abstract

Manipulating microbial communities could increase crop resistance to environmental stressors such as drought. It is, however, not clear what would be the best approach to do so and what microbial traits are important. Here, we first compare multispecies inoculums created using different approaches. The only inoculum that increased wheat fresh biomass under drought was the one created from 25 isolates that had showed a capacity to grow under high osmolarity. We then looked at two potential mechanisms of action of this inoculum: (i) direct action, by sequencing and screening the genomes of the inoculated bacteria, (ii) indirect action, by sequencing the 16S ribosomal ribonucleic acid gene and internal transcribed spacer region of rhizosphere, root, and leaves microbial communities. The microbes in the inoculum harbored many traits related to plant growth promotion, competition, and water stress resistance. The inoculation also resulted in significant shifts in the microbial communities associated with wheat, including some microorganisms (e.g. *Rhizobium*, *Shinella*, and *Klebsiella*) previously reported to improve plant drought resistance. We conclude that the inoculum studied here increased wheat growth because it potentially acted on two fronts: directly, through the traits it was selected for, and indirectly, through inducing shifts in the resident plant microbial communities.

## Introduction

Manipulating or engineering the microbiota could help crops better resist to drought [1–3]. Some microorganisms can increase the resistance of wheat to environmental stress [4–6]. Conversely, changes in soil water availability also affected the microbial communities associated with wheat [7–13]. In one of our studies, we observed that most changes in the wheat microbiota under water stress resulted from alterations in the relative abundance of already present microbes, with little recruitment from a drought-adapted multispecies inoculum or from the bulk soil [9]. This might be due to modifications in the plant exudation patterns under water stress [14] but might also be an indirect effect of the inoculation. The inoculated strains can modify the resident microbial communities, either by changing the community structure [15, 16] or its functional potential by horizontal gene transfer [17–19]. This could indirectly impact plant growth and resistance to water stress, on top of the direct effect of the inoculated microorganisms.

The inoculation of mixed microbial isolates can directly enhance crop growth in stressful conditions [20–22]. To enhance plant growth under water stress, the inoculated microbes should not only be able to resist to water stress through different mechanisms such as osmolyte accumulation, exopolysaccharides production, dehydration, dormancy, or sporulation [23, 24], but also be able to promote plant growth, through provision of nutrients and manipulation of plant hormones, among others [25–27]. Microorganisms can also increase plant drought resistance [28, 29] through manipulations of plant hormones [30, 31], provision of osmolytes [32–36] or modification of the plant epigenetics [37, 38]. Additionally, the production of secondary metabolites could enhance the competitive ability of the microorganisms, increasing the chance they will persist in the plant environment after inoculation. Clearly, a wide repertoire of traits is necessary for a microorganism to establish in the plant environment under drought and change its host phenotype.

Another factor to consider is the interactions of the inoculated microorganisms with the host—this will define their capacity to colonize and establish themselves in the plant environment. For instance, Arabidopsis plants grown in their native soil exhibited greater resistance to moderate drought than those grown in soil where corn or pine was grown [39]. Microbial communities also vary from one plant compartment to another [8, 10], resulting in different capacities to grow at low water availabilities [10]. A better understanding of the abovementioned direct and indirect mechanisms is crucial for the rational design of multi-species inoculum.

Multi-species inoculum can be created using various approaches [40]. The traditional isolation-screening approach is used to select the isolates with the most suitable traits. Although some studies have demonstrated the efficiency of this approach for inoculum creation [10, 20, 40], comparison of approaches and in-depth characterization beyond the plant responses are seldom performed. Here, we compare the effects of five different multi-species inoculums on wheat growth under normal and water stress conditions. Furthermore, we sequenced the genomes of the isolates used to develop the most effective inoculum and screened them for key traits related to life in the plant environment under water stress. We also examined the impact of the inoculation on the wheat-associated bacterial and fungal communities. This analysis provides insights into the potential mechanisms underlying the effects of multi-species inoculum on wheat growth under water stress.

## Material and methods

### Inoculum preparation

We created four multi-species inoculums and compared them to a commercial biofertilizer and a negative control. Among the four inoculums that we created, two were created by incubating agricultural soil under dry or moist conditions, and two were created using an isolate collection, by either picking the isolates most resistant to osmotic pressure or a random assortment of isolates.

### Enrichment approach (“enrich-dry” and “enrich-moist” inoculums)

Two inoculums were prepared based on the community enrichment approach [40]. Soil from our experimental field at the Armand-Frappier Santé Biotechnologie Centre (Laval, Québec, Canada) was incubated at 30°C for two months under either (i) dry conditions, in an aluminum plate without cover (“Enrich-Dry” inoculum), or (ii) moist conditions using a plastic box with a cover (“Enrich-Moist” inoculum). The moist soil was soaked with sterile water each week. The extraction of the soil microorganisms was done by suspending the soil in phosphate buffer, shaking and sonicating this suspension before centrifuging at about 100 × g, as previously recommended [41].

### Isolation-based approach (“screening” and “random” inoculums)

Two inoculums were prepared with a mixture of 25 isolates each. The “Screening” inoculum was composed of isolated that could grow under high osmotic pressure and could promote plant growth, whereas the “Random” inoculum was a random assortment of isolates from our collection. We had screened a collection of 542 isolates for growth on a hyperosmolar media containing 30% of PEG [10], which gave 44 isolates (32 bacteria, 9 fungi, and 3 unidentified isolates). These isolates were evaluated here for their potential to promote wheat germination and growth. We sterilized the surface of wheat seeds by soaking them in 70% Ethanol for 2 min and 0.5% NaOCl for 2 min, followed by five washings in sterile water [42]. We measured the percentage of germination after three days of incubation at room temperature in sterile petri dishes following inoculation with the different isolates vs. a sterile media control. We planted surface sterilized seeds in sterile soil in closed Magenta boxes before inoculating them with the microbial isolates or a sterile medium control. The boxes were placed at room temperature and watered daily with sterile water for 1 week. We categorized the germination and growth promotion capacities as positive, neutral, or negative, by comparing them with the uninoculated controls. We ended up selecting 25 isolates (23 bacteria and 2 fungi) that were able to grow under high osmotic pressure and showed positive results for germination and growth promotion assays (“Screening” inoculum). We also created a control inoculum (“Random” inoculum) by randomly selecting 25 isolates from the 542 isolates of Agoussar *et al.* [10].

Both isolate-based inoculums were prepared by mixing 1 ml (10^4^ colony forming units/ml) of each isolate growing in a liquid media (Tryptic Soy Broth for bacteria and Yeast Peptone Dextrose for fungi). The resulting 25 ml was centrifuged and cells were resuspended in sterile potassium phosphate buffer and then maintained in 25% glycerol at 4°C.

### Commercial inoculum (“biofertilizer” inoculum)

We used, as positive control, a commercial biofertilizer containing a mixture of *Mycorrhiza spp., Trichoderma spp*., and *Bacillus spp*. said to improve wheat growth under drought conditions (“Biofertilizer” inoculum). As per the provided instructions, we centrifuged 40 $\mu$l per 10 g of wheat seeds. As for the other inoculums, the pellet was resuspended with 160 $\mu$l of sterile potassium phosphate buffer and maintained in 25% glycerol at 4°C.

### Negative control (“control” inoculum)

Our negative control contained only a sterile potassium phosphate buffer and 25% glycerol. All five inoculums and the negative control were stored at 4°C until use.

### Plant growth experiment

To test the effects of our five inoculums on wheat growth under water stress, we inoculated them on wheat seeds alongside a negative control and compared wheat fresh and dry biomass when grown under low and high water availability.

The soil used in this experiment was collected from our experimental field, mixed with one-third of sand, sieved at 2 mm and autoclaved each 24 h for three successive days [43]. Wheat seeds (*Triticum aestivum)* were surface sterilized as described by Tardif *et al.* [44] and soaked in the different inoculum for one to 2 h until being seeded in the autoclaved soil. We used five seeds per pot, and three days after germination we thinned them to three plantlets per pot.

We grew the plants in a growth cabinet at 70% humidity for a cycle of 6 h of darkness at 21°C, 1 h of transition at intermediate level of luminosity (3500 lux) at 21°C, 16 h of high luminosity (8100 lux) at 25°C, and then 1 h of transition at intermediate level of luminosity at 21°C, for 4 weeks. The experiment was performed in four different growth chambers, which were considered as experimental blocks for the statistical analyses, with twelve randomly placed pots per growth chamber (two per inoculum). We watered all the pots for the first 2 weeks. For the next 2 weeks, six pots per growth chamber (one per inoculum) were maintained in normal conditions at 50% of soil water holding capacity (SWHC), while the remaining pots were maintained at 15% SWHC. We supplemented each pot with 1 ml per week of Hoagland’s nutrient solution [45].

The number of leaves was recorded weekly. At the end of the 4-week growth period, samples of roots, leaves, and rhizosphere were collected. For each sample, the length and weight of roots and leaves were measured, and the total fresh and dry weight of the plants was determined. The foliar water content was calculated as follows: (leaves fresh weight - leaves dry weight) / leaves dry weight * 100. The fresh weight was measured immediately after harvest, and the dry weight was obtained after drying the leaves at 65°C for 72 h. Based on the plant growth results obtained after 4 weeks, samples from the best performing inoculum (“Screening”) were selected together with the negative control for further analysis (total of 16 pots: 4 replicates x 2 treatments x 2 water availability).

### Deoxyribonucleic acid extraction and sequencing

Total DNA extraction was performed on 0.5 g of the wheat rhizosphere, roots, and leaves samples using the Qiagen kit (DNeasy PowerLyzer PowerSoil) (48 DNA samples: 16 pots x 3 compartments). The library preparation and MiSeq (Illumina) sequencing for 16S rRNA gene and ITS region amplicons using the primers (520F-799R) for 16S and (ITS1F-58A2R) for ITS was performed at the Centre d’expertise et de service Génome Québec (CESGQ, Montréal, Canada). We sequenced the genomes of the 23 bacterial isolates (excluding the two fungal isolates) composing the “Screening” inoculum using PacBio (for most isolates) or MiSeq (Illumina) (two isolates) at the CESGQ.

### Bioinformatic analyses

Amplicon sequencing data (16S rRNA gene and ITS region) were analyzed using AmpliconTagger [46]. Remaining high-quality reads free of sequencing adapters artifacts were dereplicated at 100% identity and clustered/denoised at 99% (DNAclust v3). Clusters of less than three reads were discarded and the remaining clusters were scanned for chimeras using UCHIME, first in de novo mode and then in reference mode [47]. The remaining clusters were clustered at 100% identity (DNAclust v3) to produce ASVs. ASVs were assigned a taxonomic lineage with the RDP classifier [48] using the Silva release 128 databases [49].

The genomic assembly for the 21 bacterial genomes sequenced by the Pacific Biosciences technology was performed at the CESGQ, while the genomic assembly for the two isolates sequenced by MiSeq was performed by SPAdes [50]. The annotation of the bacterial genome was performed based on “Prokka” Prokaryotic genome annotation on Galaxy (Version 1.14.6 + galaxy1) (http://bitly.ws/DuAz). Comparative genomic analysis of bacterial genomes was conducted using the OrthoFinder tool on Galaxy (Version 2.5.4 + galaxy1) to identify orthogroups within a collection of proteomes and to uncover conserved gene families across the 23 bacterial species. The Diamond research program was employed, and the gene tree inference method was used based on multiple sequence alignment (MSA) using the Muscle program [51], with a FastTree [52] as a tree inference method. The analysis of secondary metabolite biosynthesis gene clusters in different bacterial isolates was performed using the antiSMASH tool (version: 7.0.0beta2-86685d9d) [53].

### Statistical analysis and data visualization

Statistical analyses were performed in R (version 4.0.3, The R Foundation for Statistical Computing). If the Shapiro-Wilks and Levene tests revealed that, even after log or square root transformation, the alpha diversity and relative abundance data did not meet the assumptions for parametric analysis of variance (ANOVA), then independent one-way Kruskal–Wallis tests by rank were performed for the effects of Irrigation, Compartment, Treatment, and Block. The effect of Treatment with Screening-SynCom, Compartment and Irrigation on the bacterial and fungal community structure was visualized using principal coordinate analyses (PCoA) and tested using permutational multivariate analysis of variance (PERMANOVA) with 1000 permutations (including Blocks), both based on Bray–Curtis dissimilarity calculated from the normalized ASV tables (read counts/total read counts per sample). Differential abundance analysis was performed to compare inoculated samples against control samples across three plant compartments under drought and normal conditions. The DESeq2 package in R was used for statistical analysis, applying a negative binomial generalized linear model to account for overdispersion in the count data. Significance thresholds were set at an absolute log2 fold change ≥1 and an adjusted p-value (*P*adj) ≤ .05, corrected for multiple comparisons using the Benjamini–Hochberg method. The visualization of differentially abundant ASVs was achieved using volcano plots generated with the ggplot2 package in R. The results from plant analyses were averaged over the three plants per pot. When the effect of irrigation was significant, the analysis was then performed for the effect of Compartment, Treatment, and Block for the two irrigation regimes separately. The Dunn test was used to compare the effect of the inoculation on germination within the treatments.

## Results

### Plant leaves biomass

**Figure 1:**
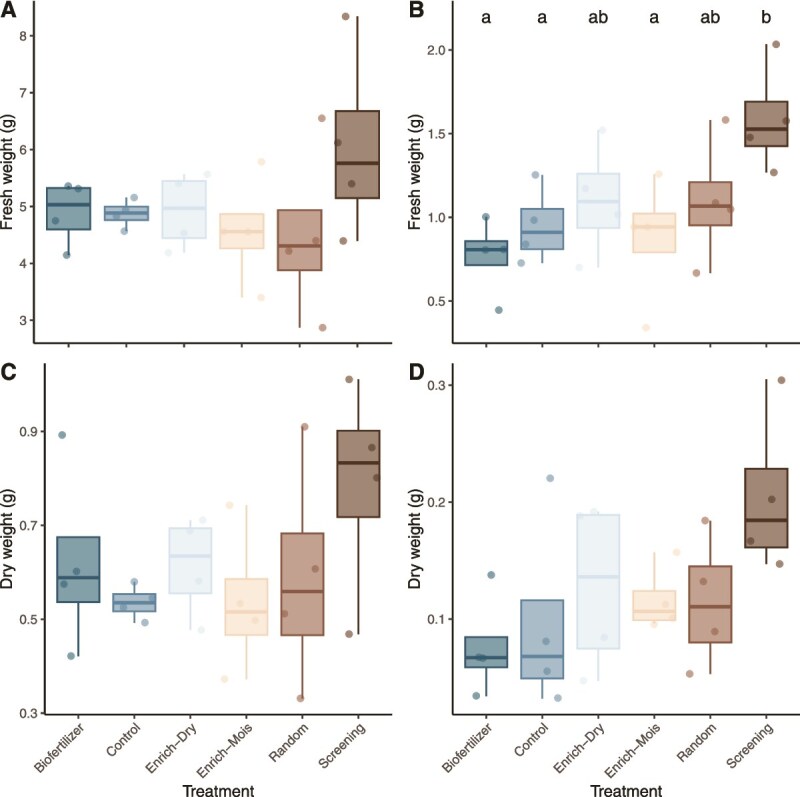
Multispecies inoculums increased plant biomass. Wheat leaves fresh weight under (A) high or (B) low SWC and wheat leaves dry weight under (C) high or (D) low SWC 4 weeks after inoculation with five different microbial communities or a sterile control. Different letters in (B) indicate significant differences between treatments (Tukey HSD test, adjusted *P* < .05).

Under low soil water content (SWC), the inoculations significantly increased leaves fresh weight (ANOVA, *P* = .0261, Fig. 1B). The plants exposed to the “screening” inoculum had a fresh weight 67.3% higher than the uninoculated control (Tukey-HSD: *P* = .08) (Fig. 1B). They were also heavier than the plants exposed to the commercial biofertilizer (Tukey-HSD: *P* = .01) and the “enrichment-moist” inoculum (Tukey-HSD: *P* = .045) (Fig. 1B). As compared to the uninoculated controls, the “screening” inoculum also increased the plant fresh weight by 24.4% under high SWC (Fig. 1A) and increased dry weight by 46.9% and 111% under high (Fig. 1C) and low SWC (Fig. 1D), respectively. These differences were, however, not statistically significant. At this point, the “screening” inoculum was the best candidate to improve wheat growth under low and high SWC and was therefore selected for further analyses.

### Microbial community structure, composition and diversity following inoculation

**Figure 2:**
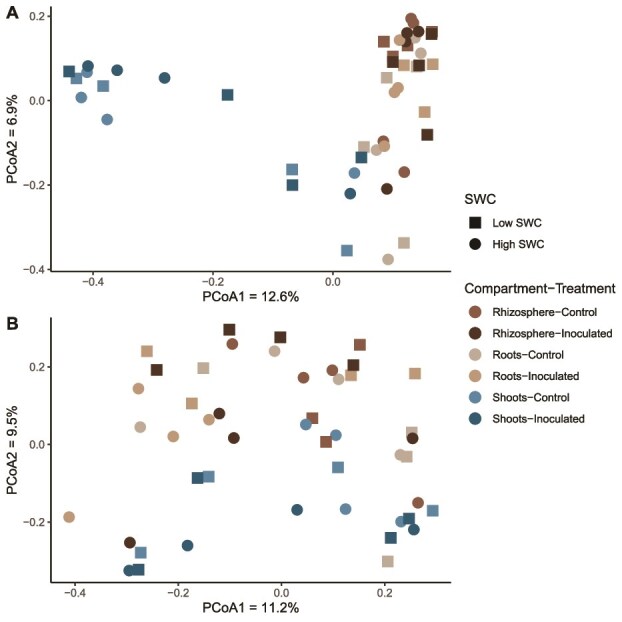
Microbial communities varied according to plant compartment, SWC and inoculation. Principal coordinates analysis of Bray–Curtis dissimilarities, calculated from the bacterial 16S rRNA gene (A) and the fungal ITS region (B) ASVs tables, for shoot root and rhizosphere samples from wheat inoculated or not with the “screening” inoculum and grown under high (50% SWHC) or low (15% SWHC) SWC.

We first looked if the “screening” inoculum modified the wheat leaves, roots, and rhizosphere microbial communities. As expected, the compartments structured microbial communities, explaining 12.3% (fungi, *P* = .001) to 20.8% (bacteria, *P* = .001) of the variation observed (Fig. 2 and Table 1). Watering explained a much smaller part of the variation, with 3.8% for bacteria (*P* = .002) and a non-significant 2.5% for fungi (*P* = .141) (Table 1). The inoculation with the “screening” consortium also significantly influenced the microbial communities, explaining 3.2% of the variation for bacteria (*P* = .015) and 4.8% for fungi (*P* = .007) (Table 1).

**Table 1:** PERMANOVA tests for the effects of inoculations, plant compartment, SWC, and their interactions on the bacterial and fungal community structure based on Bray–Curtis dissimilarities.

	Bacteria			Fungi		
	F	R^2^	P	F	R^2^	P
Compartment (C)	6.5949	0.208	**0.001** ^***^	3.2493	0.123	**0.001** ^***^
Inoculation (I)	2.0625	0.032	**0.015** ^*^	2.5568	0.048	**0.007** ^**^
SWC	2.4604	0.038	**0.002** ^**^	1.3516	0.025	0.141
Block	2.4732	0.117	**0.001** ^***^	1.3912	0.079	**0.054 .**
C:I	0.9858	0.031	0.486	0.9189	0.034	0.573
C:SWC	0.8477	0.026	0.724	0.8529	0.032	0.683
I:SWC	1.3758	0.021	0.125	1.5129	0.028	**0.084 .**
C:I:SWC	0.5440	0.017	0.996	0.5294	0.020	0.987

Values in bold are statistically significant. ^***^: *P* < .001, ^**^: .001 < *P* < .01, ^*^: .01 < *P* < .05, .: .05 < *P* < .10.

The inoculation with the “screening” consortium resulted in 9.59%, 26%, and 26% increases in bacterial Shannon diversity (*P* = .16), and Chao (*P* = .0005), and observed richness (*P* = .0003), respectively (Table 2). The bacterial Shannon diversity also varied by compartment (*P* = 9.5 × 10^−8^), but not by SWC (Table 2). Fungal alpha diversity was unaffected by the inoculation, nor any of the other experimental factors (Table 2).

**Table 2:** Statistical analysis (Kruskal–Wallis tests and ANOVA) for the effects of inoculations, plant compartment, and SWC on the bacterial and fungal alpha-diversity.

	Bacteria			Fungi		
	Shannon	Chao1	Observed	Shannon	Chao1	Observed
Compartment	**9.5 × 10** ^**−8**^ ^***^	0.49	0.47	0.58	0.82	0.82
Inoculation	0.16	**0.0005** ^***^	**0.0003** ^***^	0.65	0.68	0.68
SWC	0.56	0.58	0.52	0.20	0.82	0.82
Block	0.99	0.66	0.65	0.43	0.48	0.48

Values in bold are statistically significant. ^***^: *P* < .001.

**Figure 3:**
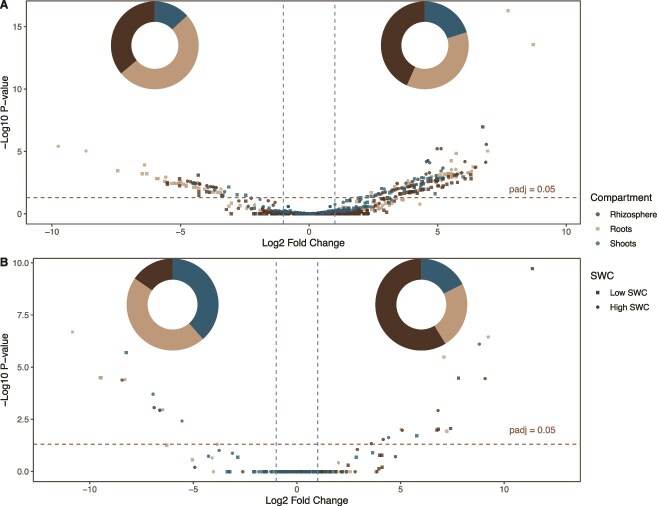
The relative abundance of many microbial ASVs is affected by inoculation. Differentially abundant bacterial (A) and fungal (B) ASVs between inoculated and non-inoculated samples. Differential abundance analyses were performed for each compartment:SWC combinations separately. Donut plots (insert) indicate the proportion for each compartment of positively (right) or negatively (left) differentially abundant ASVs that were found. ASVs with adjusted P-values below 0.05 are listed in Supplementary Tables S1–S4.

We also performed differential abundance analyses (inoculated vs. non-inoculated) at the ASV level, which we visualized using volcano plots (Fig. 3). Each combination of compartment x watering conditions was treated separately. A total of 164 bacterial ASVs showed significant positive responses to inoculation, with 80 ASVs increasing following inoculation under dry conditions and 84 under normal irrigation (Fig. 3A, Supplementary Table S1). The distribution of these positively affected ASVs across plant compartments revealed 71 ASVs (43.3%) in the rhizosphere, 60 ASVs (36.6%) in roots, and 33 ASVs (20.1%) in shoot samples. The lowest P-values were for ASVs belonging to the *Shinella* (*P* = 5.64 × 10^−17^) and *Rhizobacterium* (*P* = 2.92 × 10^−14^) genus in the root compartment under dry conditions (Fig. 3, Supplementary Table S1). Conversely, inoculation significantly decreased the relative abundance of 61 bacterial ASVs, with 38 under dry and 23 under normal conditions. The distribution of these negatively affected ASVs revealed 31 ASVs (50.8%) in roots, 22 ASVs (36.1%) in the rhizosphere, and 8 ASVs (13.1%) in shoots. The inoculation negatively affected three ASVs belonging to the *Flavobacterium* genus – two of those showed the most significant decrease among the ASVs affected, with P-values of 4.03 × 10^−6^ and 9.62 × 10^−6^ in roots under normal watering conditions (Fig. 3A, Supplementary Table S2). Three other ASVs from the same genus were positively affected by the inoculation in the rhizosphere and root samples (Supplementary Table S1). Although different isolates of the *Sphingobacterium* genus were in the inoculum, three distinct ASVs from this genus significantly decreased in relative abundance in plant roots and shoots under dry conditions (Supplementary Table S2). Conversely, the only ASV belonging to this genus that was positively affected by inoculation (ASV5) was the one matching the 16S rRNA gene of the inoculated *Sphingobacterium* isolates, which showed a significant increase in the rhizosphere under dry conditions (Supplementary Table S2). Similarly, for the *Paenibacillus* genus, four distinct ASVs decreased after 4 weeks of plant growth, while six different ASVs increased across various plant compartments under both normal and dry conditions (Fig. 3A, Supplementary Tables S1 and S2).

Inoculation positively affected 17 fungal ASVs (8 in dry and 9 in normal conditions), from which 10 ASVs were affected in the rhizosphere, 4 ASVs in the roots, and 3 ASVs in the shoots. Conversely, 13 fungal ASVs were negatively affected by the inoculation (3 under dry and 10 under normal conditions), from which 6 ASVs were affected in the roots, 5 ASVs in the shoots, and 2 ASVs in the rhizosphere (Fig. 3B, Supplementary Tables S3 and S4). The genus *Gibberella* was the most negatively affected by the inoculation, with seven different representative ASVs significantly decreasing under both normal and dry conditions across root, shoot, and rhizosphere samples. While the genus *Penicillium* was the most positively affected by the inoculation, with its increase being highly significant under both normal and dry conditions across root, shoot, and rhizosphere samples (Fig. 3B, Supplementary Table S4).

We also tested the effects of irrigation, plant compartment, and inoculation on the dominant genera found in the amplicon sequencing datasets (Supplementary Figs S1 and S2, Supplementary Table S5). We only report here the genera for which the inoculation or its interaction with another factor was significant. The *Klebsiella* was relatively more abundant in the inoculated samples (*P* = .0003), but this also interacted with irrigation (*P* = .004)—*Klebsiella* was relatively more abundant in the inoculated leaves under water stress but not so much in the inoculated leaves of well-watered plants (Supplementary Fig. S1, Supplementary Table S5). An interaction between plant compartment and inoculation was found for *Paenibacillus* (*P* = .0002). This genus was relatively less abundant in the inoculated samples under drought conditions, but not under normal watering conditions (Supplementary Fig. S1, Supplementary Table S5).

For fungi, the *Zopfiella* genus was affected by inoculation (*P* = .036), but in interaction with irrigation (*P* = .01). It was relatively more abundant in the well-watered plants and disappeared in dry plants. Additionally, for the irrigated conditions, this genus was more abundant in non-inoculated plants compared to the inoculated plants (Supplementary Fig. S2, Supplementary Table S5). The *Penicillium* genus was affected by the compartment (*P* = .001), with a higher relative abundance in inoculated roots, especially under normal watering conditions (Supplementary Fig. S2, Supplementary Table S5). Under dry conditions, the roots of inoculated plants hosted more *Humicola* (Supplementary Fig. S2) with an interactive effect of inoculation and compartment (*P* = .0003) (Supplementary Fig. S2, Supplementary Table S5). Similarly, the *Epicoccum* genus, was relatively more abundant in the inoculated rhizospheres under non-irrigated conditions compared to irrigated conditions (Supplementary Fig. S2, Supplementary Table S5) indicating an interactive effect between inoculation and plant compartments (*P* = .003).

### Genomic analysis of the inoculated microbes

We sequenced the genomes of the inoculated microorganisms. The microorganisms were identified using the average nucleotide identity (ANI) against sequenced isolates available in GenBank (Table 3). We also looked in the whole genomes for their 16S rRNA genes or ITS region and compared them to the amplicon dataset to match each inoculated microorganism to an ASV. Among the 25 inoculated strains, 5 bacteria, and 2 fungi had no match to ASVs, whereas 18 bacteria isolates had a 100% similarity with a unique ASV (Table 3). Thereby, we could determine which ASV from the inoculum potentially persisted in the plant environment and which were not detectable anymore at the time of sampling. Some of the inoculated microorganisms matched the same ASV because they were closely related and had highly similar 16S rRNA genes. They also matched the same published genome based on the ANI analysis, potentially indicating different strains from the same species (Table 3).

**Table 3:** Bacteria isolates used to prepare the “Screening” inoculum, their origin, the corresponding ASV having 100% similarity with their 16S rRNA gene, and the conditions.

Isolate	Closest match^a^	Origin of isolate^b^	ASV #	Conditions^c^
***Isolates with a match to an ASV***				
137	*Paenibacillus graminis strain DSM 15220*	DT-100%-Rhizoshpere	405	Drought-Root/Shoot
123	*P. graminis strain DSM 15220*	DS-100%-Shoot	405	Drought-Root/Shoot
351	*P. graminis strain DSM 15220*	DT-100%-Root	405	Drought-Root/Shoot
372	*Paenibacillus amylolyticus* strain SQR-21	DT-100%-Shoot	405	Drought-Root/Shoot
141	*Paenibacillus polymyxa* strain PKB1	DT-100%-Shoot	490	NS
331	*P. polymyxa* strain PKB1	DS-25%-Root	490	NS
253	Paenibacillus polysaccharolyticus, PKB1	DT-100%-Shoot	490	NS
190	*P. polymyxa* SQR-21	DS-25%-Rhizosphere	222	Drought-Root
168	*Stenotrophomonas indicatrix* strain *K279a*	DT-100%-Rhizosphere	218	Drought-Root/Shoot
300	*Bacillus pumilus*	DT-25%-Rhizosphere	644	Drought-Shoot
389	*Paenarthrobacter ilicis strain Rue61a*	DS-100%-Shoot	4	NS
376	*Paenarthrobacter ilicis strain Rue61a*	DS-100%-Rhizosphere	4	NS
417	*Arthrobacter sp.* Rue61a	DT-100%-Rhizosphere	4	NS
377	*Sphingobacterium sp. ML3W*	DT-100%-Root	15	Drought-Rhizosphere
382	*Sphingobacterium sp. ML3W*	DT-25%-Root	15	Drought-Rhizosphere
398	*Sphingobacterium sp. ML3W*	DS-100%-Shoot	15	Drought-Rhizosphere
172	*Sphingobacterium sp. ML3W*	DT-100%-Rhizosphere	15	Drought-Rhizosphere
483	*Bacillus velezensis (formerly Bacillus amyloliquefaciens subsp. plantarum NJN-6)*	DT-100%-Seed	819	NS
***Isolates that did not match any ASV***				
128	*Microbacterium sp. CGR1*	DT-25%-Shoot	NA	NA
427	*Bacillus toyonensis BCT-7112*	DS-25%-Shoot	NA	NA
411	*Bacillus toyonensis strain BCT-7112*	DT-25%- Rhizosphere	NA	NA
153	*Bacillus pseudomycoides*	DS-100%- Rhizosphere	NA	NA
276	*Microbacterium sp. No. 7*	DT-100%- Rhizosphere	NA	NA
507	*Penicillium sp. (Sanger)*	DS-100%-Shoot	NA	NA
547	*Penicillium commune (Sanger)*	DT-100%-Shoot	NA	NA

^a^Based on ANI vs. GenBank isolates.

^b^From the samples of Pande *et al.* field experiment [12]. DT: Drought tolerant wheat cultivar [*Triticum turgidum* subsp*. durum* cv. Strongfield (durum wheat)]; DS: drought sensitive wheat cultivar [*Triticum aestivum* cv AC Nass (spring wheat)]; 25% and 100%: percentage of natural precipitation. See Agoussar *et al.* [10] for more details.

^c^Experimental conditions where inoculation significantly increased ASV’s relative abundance.

None of the ASVs matching the inoculated isolates decreased in relative abundance following inoculation. Across various compartments, several of the ASVs corresponding to the inoculated isolates showed significant increase following inoculation, and this was only seen under low SWC (Table 3). For instance, ASV405, matching different isolates identified as *Paenibacillus graminis* (isolates 123, 137, 351), showed a significant increase in relative abundance in both roots and shoots under low SWC. Other ASVs, such as ASV218 and ASV644, matching the isolates *Stenotrophomonas indicatrix* and *Bacillus pumilus* respectively, showed a significant increase in relative abundance in shoots under low SWC. Similarly, ASV15, which matched different *Sphingobacterium* isolates, significantly increased in relative abundance in the rhizosphere, also under low SWC.

**Figure 4:**
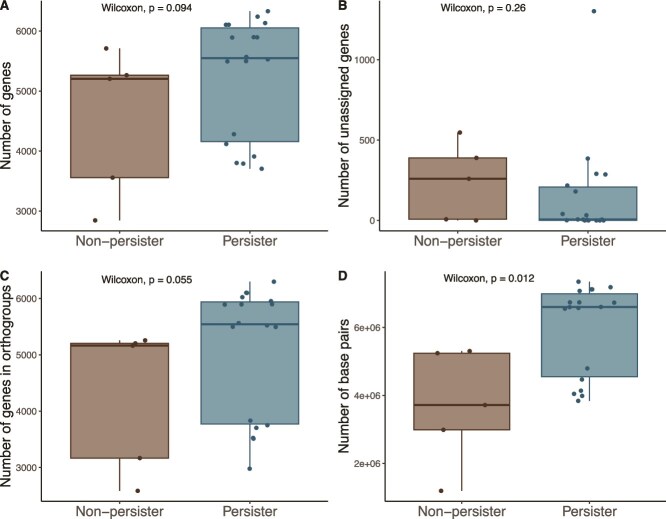
Inoculated microorganisms that persisted in the wheat environment share genomic characteristics. (A) Number of genes, (B) number of unassigned genes, (C) number of genes in orthogroups and (D) number of base pairs in the genomes of the 23 inoculated bacteria, grouped by their persistence in the wheat environment. “Persisters” are defined as the inoculated bacteria for which the 16S rRNA gene matched perfectly the 16S rRNA gene of an ASV.

Based on their detection in the plant environment at the end of the experiment, the isolates used to prepare the inoculum were separated into two groups. The first group consisted in microbes that had no match with the ASVs retrieved from the amplicon sequencing (“non-persisters”) and the second group consisted in microbes that had a match with an ASV at 100% (“persisters”). The microbes from the first group probably did not colonize the wheat environment to grow in sufficient numbers to be detectable, whereas the microbes in the second group potentially persisted in the wheat environment to reach numbers above the detection level. This comes with the caveat that the “non-persisters” might have been present, but below the detection limit of the method, whereas the “persisters” might have matched the 16S rRNA gene of a closely related ASV from the environment. With that limitation in mind, we compared the genomes of the two groups to identify potential key genomic factors implicated in persistence, but also in the larger plant biomass under water stress. As compared to the non-persisters, the potential persisters had larger genomes (*P* = .012, Fig. 4D) that contained more genes (*P* = .094, Fig. 4A), and more of these genes were part of orthogroups (*P* = .055, Fig. 4C).

**Figure 5:**
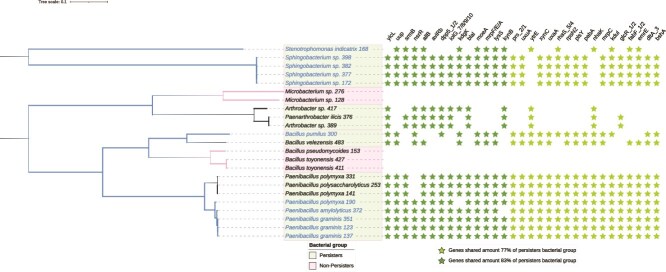
Inoculated microorganisms that persisted in the wheat environment share genes that are absent in the microorganisms that did not persist. Phylogenomic tree illustrating genetic relationships among the 23 bacterial isolates based on a comparative genomic analysis of amino acid annotations for their genomes. Branch lengths represent the genetic distances between isolates. Bacterial isolates highlighted in green persisted in the wheat environment (defined as finding a perfect match to the 16S rRNA gene of an ASV), while those in pink did not. Among the “persisters”, isolates in blue letters are those for which their corresponding ASV was significantly more abundant in the low SWC treatment compared to the high SWC treatment. Key genes shared by 14 out of 18 persisters (77%) are indicated with light green asterisks, and those shared by 15 out of 18 “persisters” (83%) are marked with dark green asterisks. In both cases the genes are absent in the “non-persisters”.

**Figure 6:**
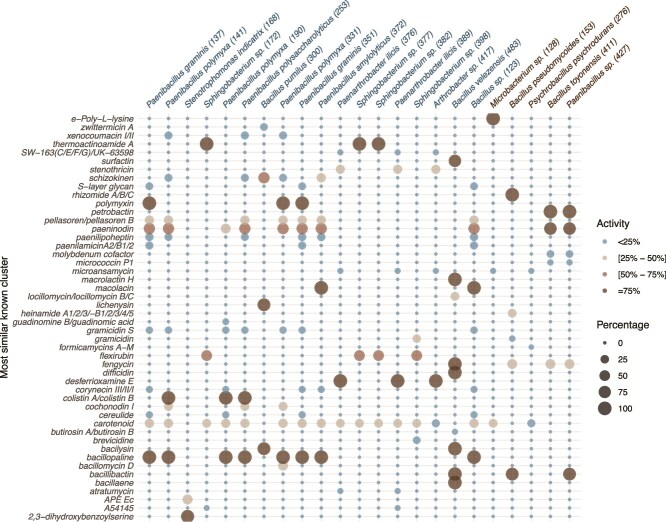
“Persisters’” genomes generally contained more secondary metabolites genes than the ones of “non-persisters”. Most similar known secondary metabolite gene clusters in the genomes of “persisters” (blue, defined as finding a perfect match to the 16S rRNA gene of an ASV) and “non-persisters” (brown).

We identified 159 genes common to the 23 bacterial strains. We also looked at the genes commonly found in the “persisters” but totally absent in the “non-persisters”. Some were shared among more than three quarters of potential “persisters” (more than 14 out of the 18 isolates) and absent in the “non-persisters” (Fig. 5). We also investigated differences between “persisters” and “non-persisters” in the presence of secondary metabolites biosynthetic gene clusters using the antiSMASH tool. We found out that some secondary metabolites gene cluster predicted for the “persisters” with similarity scores exceeds 75%, were absent among the “non-persisters” (Fig. 6). The “persisters” shared several clusters associated with antimicrobial activity, such as colistin, bacillopaline, lichenysin, macrolactin, polymyxin, and thermoactinomide A. The “non-persisters” also exhibited clusters linked to siderophore production, such as bacillibactin and petrobactin. Interestingly, the isolate *Sphingobacterium sp.* 398 did not contain any known secondary metabolite gene cluster with a similarity score above 75%, despite being categorized as a “persister”. In contrast, isolate *Bacillus velezensis* 483 contained seven known secondary metabolite gene clusters (bacillaene, bacillibactin, bacilysin, difficidin, fengycin, macrolactin, and surfactin) with similarity score above 75%.

## Discussion

Plant and soil associated microbes have a key role in helping crops adapt to abiotic stresses, such as drought [54]. One way to maximize yields under the current climatic emergency would be to manipulate the plant microbiome. Although the field is still in its infancy, a general ecological framework was suggested [3], which included migration (addition of new microorganisms) and selection (changes in the resident microbial community), two ecological mechanisms that could explain the effects of inoculants. Here, we compare these two mechanisms. We found that, for the inoculum that had worked best, both migration and selection were likely. Many genomic features of the inoculated organisms that had potentially persisted, as well as the shifts observed in the plant microbiota could explain the increased plant growth under water stress.

First, we compared inoculants that were created using different approaches. Many different methods are available to create inoculants [40], but they are rarely tested side-by-side. In a previous study, we compared microbes extracted from a soil with a water stress history or not (a naturally “evolved” inoculum), that were inoculated to wheat plant under water stress [9]. This resulted in no improvement in plant growth or water content and only modest shifts in the fungal communities in the rhizosphere [9]. We had also applied this approach in the context of bioremediation of hydrocarbon petroleum, with similar lack of improvement in plant growth and hydrocarbon degradation rates using an “evolved” inoculum [55,56]. In fact, for both studies, the control inoculum that was not “evolved” was more efficient to promote plant growth or degrade hydrocarbons [55,56]. We had suggested that a highly diversified, unselected inoculum was more prone to contain the optimal microbes for the process of interest [55,56]. Mismatches between the successional stages of the selected communities and of the inoculated ecosystem were also suspected to create problems. We also found here that the inoculum created by “evolving” soil under dry or wet conditions had little effect on wheat growth under water stress. However, in contrast to our previous studies, we did not include an unselected, highly diversified inoculum, but included instead a diversified inoculum that was created from 25 isolates that were able to grow under high osmotic pressure. This targeted approach—the “screening” inoculum—was the only one that led to improved wheat biomass under water stress.

The inoculum developed from isolates that grew under high osmotic pressure was able to promote plant growth, by increasing wheat aboveground fresh biomass under water stress, but not the dry biomass nor the root biomass. This indicates that the “Screening” inoculum probably helped wheat to retain water in its leaves. This was also evident when looking at the plant morphology, with the inoculated plants showing an improved turgor under water stress. The inoculum could have therefore stimulated stomatal closure or helped plant accumulation of osmolytes, two mechanisms that are compatible with our observations [57,58]. Simply promoting plant growth is not an ideal mechanism to help plants survive drought, because plant communities with more biomass are more susceptible to drought [48]. Our screening method, using high osmolarity growth media, selected for microbes that were highly efficient in producing osmolytes. Microbial endophytes and rhizobacteria can increase plant osmolyte concentration [33–35], and can also exude osmolytes in the plant environment [32, 36]. We also recently showed that microbial osmolytes-related transcripts were more abundant in the wheat rhizosphere when SWC decreased [12], and that intermittent water stress selected for rhizosphere microorganisms that were enriched in osmolyte producing genes [13].

Among the bacterial strains inoculated that were represented among the ASVs (the “persisters”), twelve out of seventeen were Gram-positive bacteria. Gram-positive bacteria produce osmolytes constitutively whereas Gram-negative bacteria produce them as a drought-induced response [59]. This constitutive production of osmolyte together with a thick peptidoglycan cell wall in Gram-positive bacteria allows them to remain active under low water availability, in contrast to bacteria that avoid drought by dehydration, dormancy, or sporulation [60]. Only active bacteria can protect the plant from water stress, suggesting that bacteria that resist to drought rather than avoid it would be better inoculants.

Genomic analysis of the 23 bacterial isolated used in the “screening” inoculum highlighted several genes that could be linked to the capacity of the isolate to live under low water availability. For instance, they all contained the “*BetI”* gene, responsible of choline-responsive regulator that controls the synthesis of glycine betaine, enabling osmoadaptation under hyperosmotic stress conditions [61–63]. The “*obg*” gene was also shared by the 23 isolates, and plays a key role in the bacterial stress response by helping activate the sigma B (σ^B) protein in response to environmental stress [64]. Our genomic analysis highlighted several candidate genes that could be further examined to confirm their causal role in the capacity of our isolates to live under low water availability.

Having traits related to growth under water stress is not the only thing needed for microorganisms to form a successful inoculum for helping crops resist to water stress. The microorganisms also need to establish themselves and hopefully thrive in the plant environment when they are applied. Isolates that are good candidates for improving the plant phenotype are not often screened for this trait. So, we used the differences in potential persistence among our multi-species inoculum to try to understand which genomic factors were important for successful establishment. The genomes of the “non-persisters” clustered together, and they missed several of the genes that were widely shared among the “persisters”. Many “persisters” had the genetic potential to produce different antibiotics, such as *colistin, bacillopaline, lichenysin, macolacin, polymyxin,* and *thermoactinomide A*. and other secondary metabolites, such as siderophores. The presence of these genes in the genome of the “persisters” is well aligned with the traits that are required to colonize the plant environment. For instance, the capacity of *Paenibacillus polymyxa* to promote the growth of plants was partly linked to its capability to produce polymyxin [65]. It makes sense that the potentially persistent isolates shared these traits in their genomes. These genes and the resulting traits could be targeted when screening new isolates, to increase the chance that they might establish in the plant environment. Since some of these traits might be shared by plant and human pathogens and could also be linked to antimicrobial resistance, the resulting isolates should be carefully selected.

On top of their direct effects on plants, inoculants can modify the resident soil and plant microbial communities, even when they do not persist [15, 16]. For instance, *Bacillus* can shape the microbial community in the rhizosphere [66], whereas *P. polymyxa* produces antibiotics [67], which could also modulate the microbial community. In our study, this could explain indirectly the effect of our “Screening” inoculum, as inoculation affected the bacterial and fungal communities. For example, the relative abundance of the *Shinella* genus was positively affected by the inoculation, most especially in wheat roots under water stress. Bacteria from this genus significantly enhanced duckweed biomass and root development [68] and were enriched in the rhizoplane of wheat [69]. Some strains of *Klebsiella* can accumulate osmolytes such as glycine betaine, trehalose and proline in response to drought stress [32], and, here, this genus was relatively more abundant in leaves following inoculation*.* Different genera from the Rhizobiaceae family such as *Allorhizobium*, *Neorhizobium*, *Pararhizobium*, and *Rhizobium* showed a significant increase in their relative abundance in root samples under dry conditions following the inoculation. These genera are known to increase plant osmolyte concentration [33–35] and to play a crucial role in supporting plant growth in nutrient-poor and drought-prone environments [70]. Additionally, among the nine fungal ASVs that were negatively affected by the inoculation, five of them belonged to the *Gibberella* genus, a known wheat pathogen [71,72]. Many of the inoculated bacteria had the genetic potential to produce secondary metabolites that could affect fungi. For instance, *P. polymyxa* can protect cereals against *Fusarium* head blight caused by *Fusarium culmorum* [73].

The genera listed above were not part of our inoculum and were therefore amplified from environmental strains following inoculation. More intriguing were the shifts observed in ASVs from bacterial genera that were represented in our inoculum. The ASVs matching our isolates never decreased in relative abundance following inoculation, but ASVs from the same genus were sometime negatively affected. This was the case for several ASVs from the *Paenibacillus, Sphingobacterium, Bacillus*, *Stenotrophomonas,* and *Penicillium* genera, which were well represented in our inoculum. Since these bacterial genera can enhance drought stress resistance in plants [65,74–79], and *Penicillium* can help plant accumulate osmolytes such as proline under drought [80–82], these negative effects could have reduced the positive effects of the inoculation. It would be interesting to further understand the role of niche and taxonomical overlap between the inoculated and native microorganisms on the persistence and efficiency of the inoculated microorganisms, and on its effects on the native community.

We found that, among the four approaches tested, only the inoculum made of a mixture of isolates able to grow at high osmolarity and promote plant growth successfully enhanced wheat aboveground fresh weight. Not all strains of the inoculum potentially persisted in the plant environment, and this persistence could be linked to genomic features. The question remains as to whether the inoculated strains acted directly on the plant, or indirectly through shifts in the resident microbial communities. Our data supports both mechanisms, and probably the effect on plant fresh biomass was a result of a combination of the two mechanisms. Microbiome engineering approaches that combine more than one mechanism of action are more likely to be successful [2, 3], which could provide direly needed tools to adapt crops to the ongoing climatic emergency.

## Supplementary Material

supplemental_ycaf095

## Data Availability

The genomes of the isolates are available under the NCBI BioProject accession PRJNA884320. The amplicon datasets are available under the NCBI BioProject accession PRJNA1214753.
