# Supplementary material for: Potential mode of action of multispecies inoculums on wheat growth under water stress
Source: ISME Commun. 2025 Jun 9;5(1):ycaf095. doi: 10.1093/ismeco/ycaf095 (PMC12206444; doi:10.1093/ismeco/ycaf095)

Supplemental material for :

Potential mode of action of multispecies inoculums on wheat growth under water stress

Asmaâ Agoussar[1], Julien Tremblay[1], Étienne Yergeau[1]*

[1]Centre Armand-Frappier Santé Biotechnologie, Institut national de la recherche scientifique, 531 boulevard des Prairies, Laval, QC, H7V 1B7, Canada.

Table S1: Bacterial ASVs positively affected by the inoculation

ASV	%SWHC	COMP.	BASEMEAN	LOG2FOLD CHANGE	PADJ	PHYLUM	FAMILY	GENUS
**747**	50	Root	555,731	3,327	1,37E-02	Abditibacteriota	Abditibacteriaceae	Abditibacterium
**410**	50	Rhizo	894,816	4,071	6,53E-03	Proteobacteria	Moraxellaceae	Acinetobacter
**65**	50	Rhizo	2745,752	5,745	6,79E-04	Proteobacteria	Rhizobiaceae	Allorhizob-Neorhizob-Pararhizob-Rhizobium
**65**	15	Rhizo	2366,815	5,529	3,48E-03	Proteobacteria	Rhizobiaceae	Allorhizob-Neorhizob-Pararhizob-Rhizobium
**418**	15	Shoot	1181,094	4,442	4,17E-03	Proteobacteria	Rhizobiaceae	Allorhizob-Neorhizob-Pararhizob-Rhizobium
**65**	15	Root	18124,045	8,707	2,93E-14	Proteobacteria	Rhizobiaceae	Allorhizob-Neorhizob-Pararhizob-Rhizobium
**315**	15	Root	1971,231	5,468	6,41E-04	Proteobacteria	Sphingomonadaceae	Altererythrobacter
**322**	50	Root	2442,770	5,570	1,18E-03	Proteobacteria	Sphingomonadaceae	Altererythrobacter
**315**	15	Rhizo	1377,129	4,725	2,53E-03	Proteobacteria	Sphingomonadaceae	Altererythrobacter
**549**	15	Root	748,612	3,999	3,68E-03	Proteobacteria	Sphingomonadaceae	Altererythrobacter
**549**	15	Rhizo	801,234	3,904	2,04E-02	Proteobacteria	Sphingomonadaceae	Altererythrobacter
**597**	50	Root	730,385	3,755	7,06E-03	Proteobacteria	Rhizobiaceae	Aminobacter
**597**	50	Rhizo	653,493	3,586	9,01E-03	Proteobacteria	Rhizobiaceae	Aminobacter
**163**	50	Root	1635,937	4,976	2,30E-03	Proteobacteria	Rhizobiaceae	Aureimonas
**163**	50	Shoot	1131,561	4,380	4,13E-03	Proteobacteria	Rhizobiaceae	Aureimonas
**606**	50	Rhizo	414,180	2,856	2,06E-02	Proteobacteria	Rhizobiaceae	Aureimonas
**396**	15	Root	719,509	3,937	4,07E-03	Proteobacteria	Azospirillaceae	Azospirillum
**414**	50	Root	782,778	3,862	7,06E-03	Proteobacteria	Azospirillaceae	Azospirillum
**162**	50	Root	1409,536	4,754	1,04E-03	Firmicutes	Bacillaceae	Bacillus
**644**	15	Shoot	506,998	3,131	2,01E-02	Firmicutes	Bacillaceae	Bacillus

ASV	%SWHC	COMP.	BASEMEAN	LOG2FOLD CHANGE	PADJ	PHYLUM	FAMILY	GENUS
**620**	50	Rhizo	515,559	3,211	1,34E-02	Proteobacteria	Rhizobiales Incertae Sedis	Bauldia
**793**	50	Root	365,320	2,644	2,65E-02	Proteobacteria	Rhizobiales Incertae Sedis	Bauldia
**617**	50	Rhizo	828,110	3,952	5,74E-03	Bdellovibrionota	Bdellovibrionaceae	Bdellovibrio
**291**	50	Rhizo	1219,301	4,540	6,79E-04	Actinobacteriota	Geodermatophilaceae	Blastococcus
**291**	15	Root	1164,869	4,679	1,47E-03	Actinobacteriota	Geodermatophilaceae	Blastococcus
**291**	15	Rhizo	1878,622	5,187	1,56E-03	Actinobacteriota	Geodermatophilaceae	Blastococcus
**291**	50	Root	781,388	3,859	6,36E-03	Actinobacteriota	Geodermatophilaceae	Blastococcus
**586**	15	Rhizo	864,344	4,020	1,70E-02	Proteobacteria	Beijerinckiaceae	Bosea
**406**	50	Root	1035,022	4,289	1,77E-03	Proteobacteria	Xanthobacteraceae	Bradyrhizobium
**406**	50	Rhizo	1620,166	4,965	6,29E-06	Proteobacteria	Xanthobacteraceae	Bradyrhizobium
**68**	50	Shoot	1013,837	4,213	1,77E-03	Firmicutes	Brevibacillaceae	Brevibacillus
**144**	15	Rhizo	1904,092	5,207	6,00E-03	Proteobacteria	Caulobacteraceae	Brevundimonas
**144**	50	Shoot	324,922	2,396	4,01E-02	Proteobacteria	Caulobacteraceae	Brevundimonas
**541**	50	Rhizo	953,482	4,168	4,58E-03	Proteobacteria	Caulobacteraceae	Caulobacter
**404**	50	Root	769,937	3,836	6,56E-03	Proteobacteria	Caulobacteraceae	Caulobacter
**541**	50	Root	679,902	3,644	7,58E-03	Proteobacteria	Caulobacteraceae	Caulobacter
**568**	15	Rhizo	939,123	4,147	1,52E-02	Proteobacteria	Rhodobacteraceae	Cereibacter
**568**	15	Shoot	259,792	2,001	5,01E-02	Proteobacteria	Rhodobacteraceae	Cereibacter
**212**	50	Rhizo	1232,868	4,557	6,78E-05	Bacteroidota	Chitinophagaceae	Cnuella
**464**	50	Rhizo	1631,220	4,976	2,08E-03	Firmicutes	Paenibacillaceae	Cohnella
**140**	15	Rhizo	1578,504	4,928	2,85E-03	Proteobacteria	Devosiaceae	Devosia
**403**	50	Rhizo	890,796	4,064	5,05E-03	Proteobacteria	Devosiaceae	Devosia
**111**	15	Shoot	299,124	2,251	4,35E-02	Proteobacteria	Devosiaceae	Devosia
**7**	15	Root	49889,359	5,458	7,96E-04	Bacteroidota	Spirosomaceae	Dyadobacter
**120**	50	Shoot	1676,210	4,969	1,56E-03	Bacteroidota	Spirosomaceae	Dyadobacter

ASV	%SWHC	COMP.	BASEMEAN	LOG2FOLD CHANGE	PADJ	PHYLUM	FAMILY	GENUS
**530**	15	Shoot	933,646	4,086	7,72E-03	Bacteroidota	Spirosomaceae	Dyadobacter
**7**	15	Rhizo	14423,335	5,170	7,99E-03	Bacteroidota	Spirosomaceae	Dyadobacter
**7**	15	Shoot	3092,428	3,698	1,18E-02	Bacteroidota	Spirosomaceae	Dyadobacter
**120**	50	Rhizo	5859,580	6,853	7,66E-05	Bacteroidota	Spirosomaceae	Dyadobacter
**383**	50	Root	1603,965	4,947	2,37E-03	Firmicutes	Exiguobacteraceae	Exiguobacterium
**49**	50	Shoot	1376,781	4,675	8,30E-05	Bacteroidota	Chitinophagaceae	Flaviaesturariibacter
**546**	50	Rhizo	803,111	3,905	6,66E-03	Bacteroidota	Chitinophagaceae	Flavisolibacter
**93**	15	Root	2315,883	5,707	1,49E-05	Bacteroidota	Chitinophagaceae	Flavisolibacter
**250**	50	Root	2678,056	5,705	1,18E-03	Bacteroidota	Flavobacteriaceae	Flavobacterium
**531**	50	Root	830,372	3,953	6,07E-03	Bacteroidota	Flavobacteriaceae	Flavobacterium
**16**	50	Rhizo	1556,096	3,358	4,19E-02	Bacteroidota	Flavobacteriaceae	Flavobacterium
**178**	15	Root	1900,922	5,414	6,41E-04	Proteobacteria	Kaistiaceae	Kaistia
**178**	15	Rhizo	2090,285	5,345	4,38E-03	Proteobacteria	Kaistiaceae	Kaistia
**20**	50	Shoot	2743,197	4,329	7,70E-03	Proteobacteria	Enterobacteriaceae	Klebsiella
**20**	15	Shoot	51806,008	4,886	2,47E-02	Proteobacteria	Enterobacteriaceae	Klebsiella
**475**	15	Rhizo	1289,302	4,626	1,11E-02	Actinobacteriota	Microbacteriaceae	Leifsonia
**897**	50	Shoot	402,233	2,756	2,74E-02	Actinobacteriota	Microbacteriaceae	Leucobacter
**89**	50	Rhizo	1884,980	5,190	2,25E-04	Actinobacteriota	Nocardioidaceae	Marmoricola
**191**	15	Root	2084,342	5,551	6,86E-04	Proteobacteria	Rhizobiaceae	Martelella
**191**	15	Rhizo	2179,492	5,407	1,11E-03	Proteobacteria	Rhizobiaceae	Martelella
**141**	50	Rhizo	3105,914	5,926	5,32E-04	Proteobacteria	Oxalobacteraceae	Massilia
**76**	15	Root	1971,547	5,469	6,41E-04	Proteobacteria	Oxalobacteraceae	Massilia
**43**	50	Root	2210,956	5,423	1,37E-03	Proteobacteria	Oxalobacteraceae	Massilia
**365**	50	Rhizo	1330,101	4,671	5,32E-04	Proteobacteria	Rhizobiaceae	Mesorhizobium
**295**	15	Rhizo	2529,295	5,626	8,04E-04	Proteobacteria	Beijerinckiaceae	Methylocella

ASV	%SWHC	COMP.	BASEMEAN	LOG2FOLD CHANGE	PADJ	PHYLUM	FAMILY	GENUS
**352**	15	Rhizo	1101,854	4,389	1,20E-02	Proteobacteria	Beijerinckiaceae	Methylocella
**201**	15	Root	1407,924	4,965	8,82E-04	Proteobacteria	Beijerinckiaceae	Methylorosula
**130**	15	Shoot	1426,131	4,726	1,48E-03	Proteobacteria	Beijerinckiaceae	Microvirga
**130**	50	Shoot	935,129	4,090	1,56E-03	Proteobacteria	Beijerinckiaceae	Microvirga
**394**	50	Rhizo	1655,968	4,998	2,20E-03	Proteobacteria	Beijerinckiaceae	Microvirga
**129**	15	Root	489,710	3,321	7,00E-03	Proteobacteria	Beijerinckiaceae	Microvirga
**764**	15	Rhizo	639,005	3,552	2,60E-02	Proteobacteria	Beijerinckiaceae	Microvirga
**753**	50	Rhizo	440,436	2,957	1,86E-02	Myxococcota	MyxococcalesOR	MyxococcalesOR
**152**	50	Rhizo	765,424	3,831	7,27E-03	Bacteroidota	Chitinophagaceae	Niabella
**98**	15	Shoot	1856,473	5,119	3,83E-04	Actinobacteriota	Nocardioidaceae	Nocardioides
**327**	15	Root	1856,691	5,379	6,41E-04	Actinobacteriota	Nocardioidaceae	Nocardioides
**226**	15	Rhizo	3382,849	6,053	1,56E-03	Actinobacteriota	Nocardioidaceae	Nocardioides
**312**	50	Root	1834,522	5,146	2,22E-03	Actinobacteriota	Nocardioidaceae	Nocardioides
**226**	50	Root	1427,041	4,772	2,37E-03	Actinobacteriota	Nocardioidaceae	Nocardioides
**571**	15	Rhizo	1390,178	4,739	7,99E-03	Actinobacteriota	Nocardioidaceae	Nocardioides
**639**	15	Rhizo	876,515	4,041	1,70E-02	Actinobacteriota	Nocardioidaceae	Nocardioides
**448**	15	Rhizo	1666,206	5,009	6,00E-03	Proteobacteria	Sphingomonadaceae	Novosphingobium
**60**	50	Root	3645,771	6,157	5,28E-04	Proteobacteria	Oxalobacteraceae	Oxalicibacterium
**405**	15	Shoot	1816,765	5,087	1,39E-03	Firmicutes	Paenibacillaceae	Paenibacillus
**405**	15	Root	661,028	3,803	4,08E-03	Firmicutes	Paenibacillaceae	Paenibacillus
**376**	50	Root	666,165	3,612	7,95E-03	Firmicutes	Paenibacillaceae	Paenibacillus
**725**	50	Rhizo	577,114	3,390	1,10E-02	Firmicutes	Paenibacillaceae	Paenibacillus
**647**	15	Rhizo	1052,043	4,319	1,29E-02	Firmicutes	Paenibacillaceae	Paenibacillus
**481**	15	Rhizo	751,424	3,805	1,93E-02	Firmicutes	Paenibacillaceae	Paenibacillus
**105**	50	Root	2354,493	5,515	1,18E-03	Firmicutes	Planococcaceae	Paenisporosarcina

ASV	%SWHC	COMP.	BASEMEAN	LOG2FOLD CHANGE	PADJ	PHYLUM	FAMILY	GENUS
**105**	50	Shoot	1128,205	4,375	1,26E-03	Firmicutes	Planococcaceae	Paenisporosarcina
**105**	15	Shoot	1181,779	4,443	1,39E-03	Firmicutes	Planococcaceae	Paenisporosarcina
**75**	15	Rhizo	3960,124	6,284	1,56E-03	Proteobacteria	Erwiniaceae	Pantoea
**75**	50	Rhizo	3373,994	6,048	6,78E-05	Proteobacteria	Erwiniaceae	Pantoea
**174**	50	Root	2654,997	5,692	2,84E-04	Proteobacteria	Rhodobacteraceae	Paracoccus
**174**	50	Shoot	883,405	4,003	2,65E-03	Proteobacteria	Rhodobacteraceae	Paracoccus
**174**	15	Rhizo	625,956	3,520	2,62E-02	Proteobacteria	Rhodobacteraceae	Paracoccus
**174**	50	Rhizo	5958,948	6,878	2,82E-06	Proteobacteria	Rhodobacteraceae	Paracoccus
**172**	50	Rhizo	4453,602	6,453	2,00E-04	Actinobacteriota	Solirubrobacteraceae	Patulibacter
**172**	50	Root	4035,056	6,306	4,04E-04	Actinobacteriota	Solirubrobacteraceae	Patulibacter
**172**	50	Shoot	532,669	3,213	1,72E-02	Actinobacteriota	Solirubrobacteraceae	Patulibacter
**200**	15	Rhizo	1891,796	5,198	1,56E-03	Bacteroidota	Sphingobacteriaceae	Pedobacter
**432**	50	Rhizo	589,802	3,425	3,91E-03	Bacteroidota	Sphingobacteriaceae	Pedobacter
**432**	15	Rhizo	1077,262	4,355	4,38E-03	Bacteroidota	Sphingobacteriaceae	Pedobacter
**432**	50	Root	540,988	3,284	1,18E-02	Bacteroidota	Sphingobacteriaceae	Pedobacter
**486**	15	Rhizo	814,283	3,928	1,78E-02	Bacteroidota	Sphingobacteriaceae	Pedobacter
**486**	50	Root	364,564	2,641	3,01E-02	Bacteroidota	Sphingobacteriaceae	Pedobacter
**108**	50	Root	6196,989	6,931	9,62E-06	Bacteroidota	Sphingobacteriaceae	Pedobacter
**193**	15	Root	3918,278	6,481	1,80E-04	Proteobacteria	Caulobacteraceae	Phenylobacterium
**390**	15	Rhizo	1603,096	4,952	1,56E-03	Proteobacteria	Caulobacteraceae	Phenylobacterium
**390**	50	Rhizo	1560,118	4,909	2,15E-03	Proteobacteria	Caulobacteraceae	Phenylobacterium
**340**	15	Shoot	415,716	2,808	9,66E-03	Proteobacteria	Caulobacteraceae	Phenylobacterium
**184**	15	Root	2029,597	5,512	5,76E-05	Actinobacteriota	Intrasporangiaceae	Phycicoccus
**677**	50	Rhizo	603,998	3,462	9,76E-03	Proteobacteria	Alcaligenaceae	Pigmentiphaga
**267**	50	Root	3780,534	6,210	5,28E-04	Proteobacteria	Comamonadaceae	Piscinibacter

ASV	%SWHC	COMP.	BASEMEAN	LOG2FOLD CHANGE	PADJ	PHYLUM	FAMILY	GENUS
**382**	15	Root	1896,349	5,411	6,41E-04	Firmicutes	Planococcaceae	Planococcus
**707**	50	Rhizo	539,553	3,284	1,28E-02	Proteobacteria	Caulobacteraceae	PMMR1
**707**	50	Root	402,216	2,804	2,63E-02	Proteobacteria	Caulobacteraceae	PMMR1
**131**	15	Shoot	441,428	2,907	1,06E-02	Bacteroidota	Hymenobacteraceae	Pontibacter
**157**	50	Root	2366,731	5,523	3,02E-04	Proteobacteria	Rhizobiaceae	Pseudaminobacter
**594**	50	Root	892,835	4,064	2,30E-03	Proteobacteria	Xanthobacteraceae	Pseudolabrys
**594**	50	Rhizo	376,493	2,699	2,46E-02	Proteobacteria	Xanthobacteraceae	Pseudolabrys
**397**	15	Root	1522,756	5,083	7,96E-04	Proteobacteria	Pseudomonadaceae	Pseudomonas
**169**	50	Shoot	1962,749	5,203	1,33E-03	Proteobacteria	Pseudomonadaceae	Pseudomonas
**2**	15	Rhizo	56946,432	4,398	2,85E-03	Proteobacteria	Pseudomonadaceae	Pseudomonas
**397**	50	Root	1097,859	4,378	3,89E-03	Proteobacteria	Pseudomonadaceae	Pseudomonas
**169**	50	Root	804,434	3,904	6,33E-03	Proteobacteria	Pseudomonadaceae	Pseudomonas
**115**	15	Root	2572,522	5,863	5,80E-04	Firmicutes	Planococcaceae	Psychrobacillus
**55**	50	Shoot	1624,180	4,922	8,99E-04	Proteobacteria	Comamonadaceae	Ramlibacter
**565**	15	Rhizo	789,064	3,880	1,93E-02	Gemmatimonadota	Gemmatimonadaceae	Roseisolibacter
**77**	50	Rhizo	1279,852	4,613	2,74E-03	Proteobacteria	Rhizobiaceae	Shinella
**145**	15	Rhizo	2766,177	5,758	3,15E-03	Proteobacteria	Rhizobiaceae	Shinella
**77**	15	Shoot	726,548	3,699	9,66E-03	Proteobacteria	Rhizobiaceae	Shinella
**77**	15	Rhizo	5410,275	6,739	1,13E-07	Proteobacteria	Rhizobiaceae	Shinella
**77**	15	Root	9187,067	7,723	5,65E-17	Proteobacteria	Rhizobiaceae	Shinella
**160**	50	Shoot	922,961	4,070	7,37E-03	Bacteroidota	Cytophagaceae	Siphonobacter
**208**	50	Root	1156,278	4,456	3,54E-03	Firmicutes	Planococcaceae	Solibacillus
**208**	50	Shoot	753,111	3,757	3,64E-03	Firmicutes	Planococcaceae	Solibacillus
**329**	15	Rhizo	1152,793	4,457	1,11E-02	Proteobacteria	Sphingomonadaceae	Sphingoaurantiacus
**264**	50	Root	579,660	3,393	3,54E-03	Bacteroidota	SphingobacterialesOR	SphingobacterialesOR

ASV	%SWHC	COMP.	BASEMEAN	LOG2FOLD CHANGE	PADJ	PHYLUM	FAMILY	GENUS
**264**	50	Rhizo	1796,415	5,119	6,29E-06	Bacteroidota	SphingobacterialesOR	SphingobacterialesOR
**15**	15	Rhizo	21435,181	4,199	2,35E-02	Bacteroidota	Sphingobacteriaceae	Sphingobacterium
**205**	15	Rhizo	839,251	3,975	1,72E-02	Proteobacteria	Sphingomonadaceae	Sphingopyxis
**218**	15	Root	3634,363	6,371	1,80E-04	Proteobacteria	Xanthomonadaceae	Stenotrophomonas
**218**	15	Shoot	3344,711	5,987	1,14E-03	Proteobacteria	Xanthomonadaceae	Stenotrophomonas
**351**	50	Root	1533,310	4,880	2,32E-03	Actinobacteriota	Streptomycetaceae	Streptomyces
**351**	15	Shoot	364,292	2,588	3,18E-02	Actinobacteriota	Streptomycetaceae	Streptomyces
**937**	50	Shoot	350,466	2,525	3,66E-02	Actinobacteriota	Streptomycetaceae	Streptomyces
**596**	15	Shoot	1322,889	4,613	1,62E-03	Bacteroidota	Chitinophagaceae	Taibaiella
**230**	15	Rhizo	3920,978	6,269	7,50E-04	Proteobacteria	Xanthomonadaceae	Thermomonas
**253**	15	Root	1826,724	5,355	8,82E-04	Firmicutes	Alicyclobacillaceae	Tumebacillus
**222**	15	Root	1696,804	5,245	6,89E-04	Firmicutes	Planococcaceae	uncultured-Planococcaceae
**645**	50	Root	769,681	3,836	2,32E-03	Proteobacteria	Xanthobacteraceae	uncultured-Xanthobacteraceae
**284**	15	Rhizo	2667,184	5,704	8,04E-04	Proteobacteria	Alcaligenaceae	Verticiella
**284**	15	Root	704,247	3,903	3,89E-03	Proteobacteria	Alcaligenaceae	Verticiella
**621**	15	Root	287,861	2,413	2,05E-02	Myxococcota	NA	
**371**	15	Rhizo	1653,533	4,998	6,00E-03	Proteobacteria	Rhizobiaceae	
**660**	15	Rhizo	701,237	3,697	2,20E-02	Proteobacteria	Beijerinckiaceae	
**554**	50	Rhizo	1282,364	4,616	5,52E-05	Bacteroidota	Sphingobacteriaceae	

Table S2: Bacterial ASVs negatively affected by the inoculation

ASV	%SWHC	COMP.	BASEMEAN	LOG2FOLD CHANGE	PADJ	PHYLUM	FAMILY	GENUS
**410**	15	Rhizo	1043,627	-4,307	8,04E-04	Proteobacteria	Moraxellaceae	Acinetobacter
**417**	15	Shoot	1230,766	-4,505	3,82E-03	Actinobacteriota	Nocardioidaceae	Aeromicrobium
**323**	50	Rhizo	717,049	-3,730	7,35E-03	Proteobacteria	Xanthobacteraceae	Afipia
**303**	15	Root	2419,471	-5,366	3,85E-03	Proteobacteria	Rhizobiaceae	Allorhizob-Neorhizob-Pararhizob-Rhizobium
**354**	15	Root	1342,635	-4,495	7,58E-03	Proteobacteria	Sphingomonadaceae	Altererythrobacter
**510**	50	Rhizo	665,929	-3,615	8,09E-03	Proteobacteria	Sphingomonadaceae	Altererythrobacter
**414**	15	Rhizo	779,274	-3,861	1,93E-02	Proteobacteria	Azospirillaceae	Azospirillum
**414**	15	Root	676,583	-3,458	2,11E-02	Proteobacteria	Azospirillaceae	Azospirillum
**477**	15	Root	793,980	-3,703	1,81E-02	Actinobacteriota	Brevibacteriaceae	Brevibacterium
**378**	50	Root	1720,256	-5,051	2,32E-03	Proteobacteria	Caulobacteraceae	Brevundimonas
**378**	50	Rhizo	1168,547	-4,476	3,39E-03	Proteobacteria	Caulobacteraceae	Brevundimonas
**285**	15	Rhizo	1697,682	-5,037	1,56E-03	Proteobacteria	Caulobacteraceae	Caulobacter
**179**	15	Root	10003,495	-7,433	3,62E-04	Bacteroidota	Chitinophagaceae	Chitinophaga
**516**	15	Root	1922,358	-5,027	3,90E-03	Bacteroidota	Chitinophagaceae	Chitinophaga
**585**	15	Root	1171,008	-4,291	9,18E-03	Bacteroidota	Weeksellaceae	Chryseobacterium
**268**	50	Rhizo	1282,976	-4,617	2,74E-03	Firmicutes	Paenibacillaceae	Cohnella
**471**	15	Root	1979,996	-5,071	3,90E-03	Bacteroidota	Spirosomaceae	Dyadobacter
**90**	15	Rhizo	1029,453	-4,286	1,78E-02	Bacteroidota	Flavobacteriaceae	Flavobacterium
**48**	50	Root	43377,874	-9,748	4,04E-06	Bacteroidota	Flavobacteriaceae	Flavobacterium
**88**	50	Root	20589,229	-8,671	9,62E-06	Bacteroidota	Flavobacteriaceae	Flavobacterium
**151**	15	Root	2847,179	-5,604	2,61E-03	Proteobacteria	Kaistiaceae	Kaistia
**151**	15	Shoot	506,209	-3,129	7,34E-03	Proteobacteria	Kaistiaceae	Kaistia

ASV	%SWHC	COMP.	BASEMEAN	LOG2FOLD CHANGE	PADJ	PHYLUM	FAMILY	GENUS
**151**	15	Rhizo	841,766	-3,979	1,72E-02	Proteobacteria	Kaistiaceae	Kaistia
**470**	15	Root	2240,462	-5,253	3,59E-03	Proteobacteria	Comamonadaceae	Limnohabitans
**310**	15	Root	5217,075	-6,488	6,41E-04	Proteobacteria	Xanthomonadaceae	Lysobacter
**70**	50	Rhizo	1383,724	-4,730	2,51E-03	Proteobacteria	Oxalobacteraceae	Massilia
**234**	15	Root	1212,098	-4,342	3,36E-03	Proteobacteria	Beijerinckiaceae	Methylobacterium-Methylorubrum
**234**	50	Rhizo	842,548	-3,979	5,64E-03	Proteobacteria	Beijerinckiaceae	Methylobacterium-Methylorubrum
**416**	50	Rhizo	1019,167	-4,269	3,91E-03	Proteobacteria	Beijerinckiaceae	Microvirga
**447**	50	Rhizo	1130,113	-4,426	3,91E-03	Bacteroidota	Spirosomaceae	Nibrella
**296**	15	Rhizo	2354,231	-5,521	3,41E-03	Proteobacteria	Oxalobacteraceae	Noviherbaspirillum
**358**	50	Root	590,750	-3,423	1,06E-02	Proteobacteria	Sphingomonadaceae	Novosphingobium
**102**	50	Root	2447,534	-5,573	1,18E-03	Actinobacteriota	Micrococcaceae	Paenarthrobacter
**135**	15	Root	3411,613	-5,869	1,54E-03	Firmicutes	Paenibacillaceae	Paenibacillus
**331**	50	Rhizo	1206,483	-4,524	3,49E-03	Firmicutes	Paenibacillaceae	Paenibacillus
**566**	50	Rhizo	717,173	-3,730	7,27E-03	Firmicutes	Paenibacillaceae	Paenibacillus
**204**	15	Shoot	337,563	-2,459	3,27E-02	Firmicutes	Paenibacillaceae	Paenibacillus
**425**	50	Root	665,936	-3,611	8,48E-03	Proteobacteria	Rhodobacteraceae	Paracoccus
**412**	50	Root	1643,950	-4,983	2,67E-03	Actinobacteriota	Solirubrobacteraceae	Patulibacter
**412**	50	Rhizo	1006,231	-4,250	4,42E-03	Actinobacteriota	Solirubrobacteraceae	Patulibacter
**199**	50	Root	1092,974	-4,371	3,84E-03	Bacteroidota	Sphingobacteriaceae	Pedobacter
**199**	15	Rhizo	2336,300	-5,509	4,38E-03	Bacteroidota	Sphingobacteriaceae	Pedobacter
**514**	15	Rhizo	1698,333	-5,037	6,02E-03	Bacteroidota	Sphingobacteriaceae	Pedobacter
**400**	15	Root	722,523	-3,559	1,96E-02	Bacteroidota	Sphingobacteriaceae	Pedobacter
**123**	15	Root	1257,327	-4,397	7,68E-03	Proteobacteria	Caulobacteraceae	Phenylobacterium

ASV	%SWHC	COMP.	BASEMEAN	LOG2FOLD CHANGE	PADJ	PHYLUM	FAMILY	GENUS
**12**	50	Shoot	845,667	-3,936	8,15E-03	Proteobacteria	Pseudomonadaceae	Pseudomonas
**31**	50	Shoot	714,175	-3,675	1,29E-02	Proteobacteria	Comamonadaceae	Ramlibacter
**332**	15	Root	1007,955	-4,065	1,08E-02	Proteobacteria	Acetobacteraceae	Roseomonas
**332**	15	Rhizo	1797,784	-4,285	3,21E-02	Proteobacteria	Acetobacteraceae	Roseomonas
**160**	15	Root	3526,485	-5,917	1,54E-03	Bacteroidota	Cytophagaceae	Siphonobacter
**160**	15	Shoot	414,330	-2,803	2,99E-02	Bacteroidota	Cytophagaceae	Siphonobacter
**36**	15	Root	16241,027	-6,392	1,24E-04	Bacteroidota	Sphingobacteriaceae	Sphingobacterium
**552**	15	Root	1633,037	-4,786	6,22E-03	Bacteroidota	Sphingobacteriaceae	Sphingobacterium
**36**	15	Shoot	2407,438	-3,402	3,55E-02	Bacteroidota	Sphingobacteriaceae	Sphingobacterium
**177**	15	Root	1761,278	-4,898	4,39E-03	Proteobacteria	Sphingomonadaceae	Sphingobium
**624**	15	Rhizo	564,984	-3,359	3,21E-02	Proteobacteria	Sphingomonadaceae	Sphingomonas
**182**	15	Root	1631,074	-4,784	5,18E-03	Proteobacteria	Sphingomonadaceae	Stakelama
**46**	50	Shoot	557,949	-3,286	1,72E-02	Proteobacteria	Xanthomonadaceae	Stenotrophomonas
**343**	15	Root	2110,093	-5,164	3,85E-03	Bacteroidota	Chitinophagaceae	Taibaiella
**253**	50	Rhizo	767,422	-3,835	6,88E-03	Firmicutes	Alicyclobacillaceae	Tumebacillus
**256**	15	Root	4596,629	-6,304	6,41E-04	Proteobacteria	Comamonadaceae	Xylophilus

Table S3: Fungal ASVs positively affected by the inoculation

ASV	SWC	COMPART	BASEMEAN	LOG2FOLD CHANGE	PADJ	PHYLUM	FAMILY	GENUS
**71**	50	Rhizo	2012,750	6,792	1,16E-03	Ascomycota	SordarialesfamIncertaesedis	Staphylotrichum
**64**	15	Rhizo	3475,857	7,394	8,47E-03	Basidiomycota	Trichosporonaceae	Cutaneotrichosporon
**37**	15	Rhizo	2316,857	6,806	9,33E-03	Ascomycota	Aspergillaceae	Aspergillus
**71**	50	Root	598,875	5,012	9,39E-03	Ascomycota	SordarialesfamIncertaesedis	Staphylotrichum
**25**	15	Rhizo	2214,571	6,740	1,00E-02	Ascomycota	NA	NA
**7**	50	Rhizo	622,750	5,070	1,03E-02	Ascomycota	Pleosporaceae	Bipolaris
**23**	15	Root	15467,500	7,214	1,13E-02	Ascomycota	HypocrealesfamIncertaesedis	Sarocladium
**57**	15	Shoot	3244,250	5,773	1,88E-02	Ascomycota	Cladosporiaceae	Cladosporium
**147**	50	Shoot	401,375	4,413	2,30E-02	Basidiomycota	Punctulariaceae	Punctularia
**123**	50	Rhizo	338,000	4,152	2,84E-02	Ascomycota	Aspergillaceae	Aspergillus
**39**	50	Rhizo	233,875	3,584	4,52E-02	Ascomycota	Aspergillaceae	Aspergillus
**16**	15	Rhizo	53557,286	11,346	1,89E-10	Ascomycota	Aspergillaceae	Penicillium
**16**	15	Root	2444,000	7,074	3,23E-06	Ascomycota	Aspergillaceae	Penicillium
**8**	15	Rhizo	53580,857	7,778	3,28E-05	Ascomycota	Nectriaceae	Gibberella
**16**	50	Rhizo	9642,875	9,063	3,48E-05	Ascomycota	Aspergillaceae	Penicillium
**11**	50	Root	10712,000	9,215	3,51E-07	Ascomycota	Aspergillaceae	Penicillium
**16**	50	Shoot	7953,000	8,784	7,77E-07	Ascomycota	Aspergillaceae	Penicillium

Table S4: Fungal ASVs negatively affected by the inoculation

ASV	%SWHC	COMP.	BASEMEAN	LOG2FOLD CHANGE	PADJ	PHYLUM	FAMILY	GENUS
**35**	50	Shoot	2217,500	-6,933	1,94E-04	Ascomycota	Nectriaceae	Gibberella
**35**	50	Rhizo	2140,625	-6,882	8,45E-04	Ascomycota	Nectriaceae	Gibberella
**28**	50	Root	1608,125	-6,465	1,06E-03	Ascomycota	Chaetomiaceae	Humicola
**26**	50	Shoot	1783,375	-6,616	1,13E-03	Ascomycota	Nectriaceae	Gibberella
**33**	50	Shoot	1763,750	-6,600	1,13E-03	Ascomycota	Nectriaceae	Gibberella
**95**	50	Shoot	859,250	-5,546	3,73E-03	Ascomycota	Aspergillaceae	Penicillium
**1**	50	Root	110460,375	-3,842	4,88E-02	Ascomycota	Chaetomiaceae	Ovatospora
**35**	15	Shoot	5399,875	-8,224	1,97E-06	Ascomycota	Nectriaceae	Gibberella
**26**	50	Root	32560,125	-10,820	2,03E-07	Ascomycota	Nectriaceae	Gibberella
**2**	15	Root	47153,500	-9,441	3,19E-05	Ascomycota		NA
**49**	15	Root	12933,625	-9,487	3,19E-05	Ascomycota	Nectriaceae	Gibberella
**37**	50	Root	5620,250	-8,282	3,90E-05	Ascomycota	Aspergillaceae	Aspergillus
**37**	50	Rhizo	6201,500	-8,424	4,10E-05	Ascomycota	Aspergillaceae	Aspergillus

Table S5. The effects of irrigation, plant compartment and inoculation on the dominant genera found in the amplicon sequencing datasets based on Kruskal-Wallis test with Benjamini-Hochberg Adjusted p-values.

	Compartment (C)	inoculation (I)	SWC	block
Bacteria				
Flavobacterium	**P=0.008**<br>P(adj)=0.028***	P=0.749<br>P(adj)=0.873	**P=0.001**<br>P(adj)=0.007****	**P=0.070.**<br>P(adj)=0.148
Klebsiella	P=0.849<br>P(adj)=0.849	**P=0.0003***<br>P(adj)=0.002****	P=0.966<br>P(adj)=1	P=0.667<br>P(adj)=0.667
Bacillus	**P=0.063.<br>P(adj)=0.091.**	P=0.456<br>P(adj)=0.798	P=0 .394<br>P(adj)=1	**P=0.076.**<br>P(adj)=0.138
Paenibacillus	**P =0.0002***<br>P(adj)=0.001***	P= 0.209<br>P(adj)=0.646	P=0.609<br>P(adj)=1	P=0.537<br>P(adj)=0.626
Stenotrophomonas	**P=0.068.<br>P(adj)=0.091.**	P= 1<br>P(adj)=1	P =0.670<br>P(adj)=1	**P=0.084.**<br>P(adj)=0.138
Sphingobacterium	**P =0.039*<br>P(adj)=0.091.**	P=0.686<br>P(adj)=0.873	P =1<br>P(adj)=1	**P=0.009**<br>P(adj)=0.063.**
Fungi				
Zopfiella	P=0.570<br>P(adj)=0.57	**P=0.004**<br>P(adj)=0.036***	P=0.147<br>P(adj)=0.436	**P=0.070**<br>P(adj)=0.171
Epicoccum	**P=0.003**<br>P(adj)=0.009****	P=0.798<br>P(adj)=0.897	P=0.848<br>P(adj)=0.983	P=0.45<br>P(adj)=0.578
Aspergillus	P=0.318<br>P(adj)=0.357	P=0.317<br>P(adj)= 0.709	**P=0.028***<br>P(adj)=0.252	**P=0.076.**<br>P(adj)=0.171
Penicilium	**P=0.0002***<br>P(adj)=0.001****	P=0.317<br>P(adj)= 0.709	P=0.983<br>P(adj)=0.983	**P=0.097.**<br>P(adj)=0.174
Humicola	**P=0.0003***<br>P(adj)=0.001****	P=0.966<br>P(adj)= 0.966	P=0.443<br>P(adj)=0.797	**P=0.066.**<br>P(adj)=0.171
Papulaspora	P=0.172<br>P(adj)=0.258	**P=0.041***<br>P(adj)= 0.184	P=0.551<br>P(adj)=0.826	P=0.707<br>P(adj)=0.707

∗∗∗: P < 0.001, ∗∗: 0.001 < P < 0.01, ∗:0.01 < P < 0.05, .: 0.05 < P < 0.10

Figure S1. Relative abundance of the dominant bacterial genera (on average, more than 1% of the 16S rRNA gene reads) retrieved from shoot, root and rhizosphere samples from wheat inoculated or not with and growing under two different soil water content (15% and 50% SWHC).

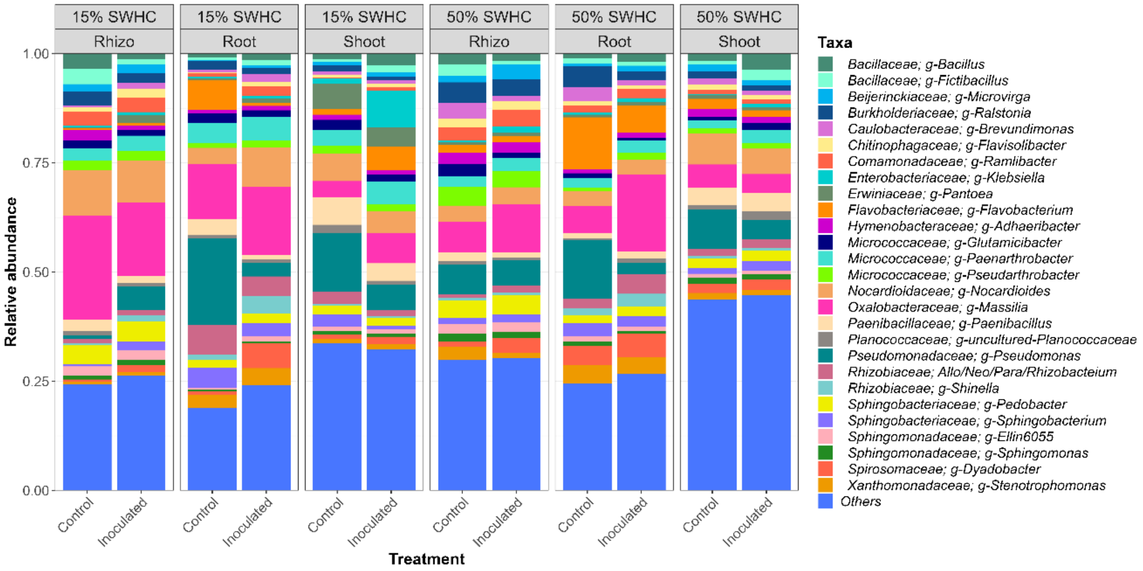

Figure S2. Relative abundance of the dominant fungal genera (on average, more than 1% of the ITS reads) retrieved from shoot, root and rhizosphere samples from wheat inoculated or not with and growing under two different soil water content (15% and 50% SWHC).

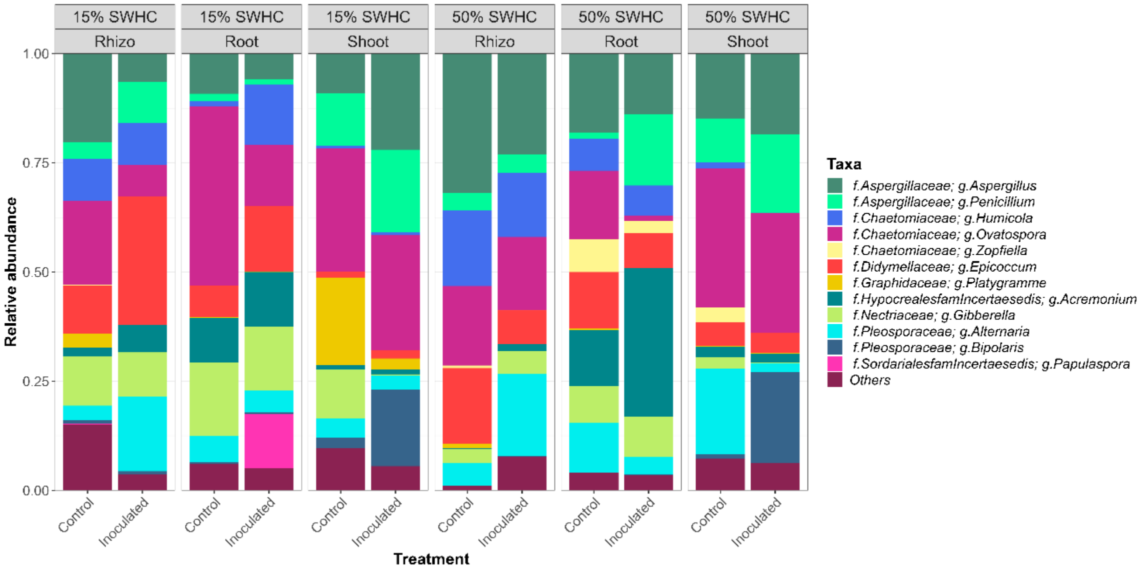

Supplement: supplemental_ycaf095 [file supplemental_ycaf095.pdf]
